# ICAM-1 related long noncoding RNA is associated with progression of IgA nephropathy and fibrotic changes in proximal tubular cells

**DOI:** 10.1038/s41598-022-13521-6

**Published:** 2022-06-10

**Authors:** Lu Wen, Zhanzheng Zhao, Fanghua Li, Fengping Ji, Jianguo Wen

**Affiliations:** 1 Department of Nephrology, The First Affiliated Hospital of Zhengzhou University, Zhengzhou, 450052 China; 2 Department of Urology, The First Affiliated Hospital of Zhengzhou University, Zhengzhou, 450052 China; 3 Henan Joint International Pediatric Urodynamic Center, The First Affiliated Hospital of Zhengzhou University, Zhengzhou, 450052 China

**Keywords:** Chronic kidney disease, End-stage renal disease, IgA nephropathy, Renal fibrosis, Predictive markers, Prognostic markers

## Abstract

Intercellular adhesion molecule 1 (ICAM-1) related long noncoding RNA (ICR) is on the antisense strand of ICAM-1 and regulates ICAM-1 expression. ICAM-1 is involved in renal tubulointerstitial injury; however, the expression and clinical implication of ICR are not determined in IgA nephropathy (IgAN). We compared renal ICR levels in 337 IgAN patients with those of 89 biopsy controls, and a markedly increased ICR level was observed in IgAN patients. By Cox proportional hazards models, higher levels of renal ICR were independently associated with disease progression event defined as end-stage renal disease or ≥ 40% decline in estimated glomerular filtration rate. Patients in the highest tertile of renal ICR had a 3.5-fold higher risk for disease progression compared with those in the lowest tertile. The addition of renal ICR to a model with traditional risk factors improved risk prediction of disease progression (net reclassification index: 0.31 [95% CI 0.01–0.50]; integrated discrimination index: 0.10 [95% CI 0.04–0.16]). Inhibition of ICR by transfection with plasmids containing ICR shRNA significantly reduced expression of collagen I and α-SMA, and phosphorylation of Akt and mTOR in TGF-β1- treated HK-2 cells. Our findings suggest that renal ICR might be an independent predictor of IgAN progression and contribute to renal fibrosis.

## Introduction

IgA nephropathy (IgAN) is one of the most common primary glomerulonephritis worldwide^1, 2^, and is characterized by the predominant deposits of IgA in the glomerular mesangium associated with increased mesangial matrix and hypercellularity^2^. Approximately 15–40% of IgAN patients will progress to end-stage renal disease (ESRD) within 20 years of onset^3^. Thus, the identification of the risk factors associated with disease progression is essential in patients with IgAN. Accumulating evidence indicates that some clinical parameters, such as reduced estimated glomerular filtration rate (eGFR), increased urinary protein excretion, hypertension and severe histological grade, are independent risk factors for IgAN progression^4–6^. Recently, numerous biomarkers, including noncoding RNAs, have been observed to participate in and predict the development of IgAN^7–10^.

Long noncoding RNAs (lncRNAs) are a type of RNA with a length of > 200 nucleotides that do not produce proteins^11^. LncRNAs can modulate gene expression at both transcriptional and posttranscriptional levels by changing chromatin states, regulating transcription factors, or hosting other small RNAs^12^. Emerging data have suggested important roles of lncRNAs in pathogenesis and disease progression of IgAN^13, 14^.

Intercellular adhesion molecule 1 (ICAM-1) related lncRNA (ICR) is transcribed from the anti-sense DNA strand overlapping the ICAM1–ICAM4–ICAM5 gene cluster, and is 3.22 Kb long with a single exon that begins in the ICAM4–ICAM5 intergenic region and overlaps the 3′ untranslated region of gen ICAM1^15^. ICR has been shown to bind to and stabilize the ICAM-1 transcript, causing increased ICAM-1 protein expression in hepatocellular carcinoma^16^. Moreover, renal proximal tubule ICAM-1 is identified to play an essential role in tubulointerstitial injury associated with TGF-β1 generation and fibrotic changes in chronic kidney disease (CKD)^17, 18^. Thus, the present study investigated the renal expression of ICR and its potential relationship with disease progression in patients with IgAN, based on the hypothesis that renal ICR may regulate ICAM-1 expression in kidney.

In this study, we first measured ICR level at baseline in kidney biopsy tissue from 337 patients with IgAN using quantitative PCR (qPCR), and evaluated the relationship between renal ICR and disease progression in IgAN. In addition, we analyzed whether addition of ICR level to the traditional risk factors could improve risk assessment for IgAN progression. We further detected ICR expression in human proximal tubular HK-2 cells in vitro, and examined the effects of ICR on fibrotic changes in HK-2 cells upon transforming growth factor-β 1 (TGF-β1) treatment.

## Results

### Demographic, clinical and histological data of IgAN patients

Demographic, clinical and histological data of the IgAN patients as well as measurements of renal ICR are summarized in Table 1. A total of 337 IgAN patients were recruited in this study. Of these, 193 (57.3%) were male and 144 (42.7%) female, with a median age of 35.0 (interquartile range [IQR] 26.5–44.0) years. At the time of biopsy, the median eGFR, urinary protein excretion and mean arterial pressure (MAP) were 83.2 (IQR 58.2–108.2) mL/min/1.73m^2^, 1.48 (IQR 0.83–2.77) g/24 h and 99.0 (IQR 93.0–107.1) mmHg, respectively. With regard to Oxford pathological lesions, the percentages of IgAN patients with M1, E1 and S1 were 43.0%, 29.7% and 57.6%, respectively; the percentages of patients with T1 and T2 were 33.2% and 21.7%, while the percentages of patients with C1 and C2 were 43.0% and 6.5%. The median follow-up time of all patients was 42.96 (IQR 37.31–61.35) months. Overall, 181 (53.7%) patients received steroids or other immunosuppressive agents. There were 102 (30.3%) patients reaching the composite disease progression event which included 20 (5.9%) ESRD events.

**Table 1:** Characteristics of IgA nephropathy patients by renal ICR tertiles at biopsy.

Variables	Overall (*n* = 337)	Renal ICR			*P*
		Group 1 1st tertile (*n* = 112)	Group 2 2nd tertile (*n* = 113)	Group 3 3rd tertile (*n* = 112)	
**Baseline**					
Renal ICR levels	2.11 (1.21–4.27)	< 1.56	1.56–3.04	> 3.04	< 0.001
Age (years)	35.0 (26.5–44.0)	33.0 (28.0–46.0)	32.0 (23.0–45.5)	36.0 (29.2–43.0)	0.075
Sex (men, %)	193 (57.3)	57 (50.9)	75 (66.4)	61 (54.5)	0.049
MAP (mmHg)	99.0 (93.0–107.1)	99.5 (93.0–105.5)	95.6 (90.3–103.8)	104.5 (97.0–112.6)	< 0.001
eGFR (mL/min/1.73 m^2^)	83.2 (58.2–108.2)	91.3 (55.9–111.6)	78.7 (60.8–108.3)	80.9 (50.2–105.3)	0.157
Proteinuria (g/24 h)	1.48 (0.83–2.77)	1.22 (0.77–2.15)	1.42 (0.92–2.55)	1.88 (0.81–3.15)	0.006
**Oxford classification**					
M1, n (%)	145 (43.0)	32 (28.6)	48 (42.5)	65 (58.0)	< 0.001
E1, n (%)	100 (29.7)	30 (26.8)	34 (30.1)	36 (32.1)	0.676
S1, n (%)	194 (57.6)	40 (35.7)	83 (73.5)	71 (63.4)	< 0.001
T1, n (%)	112 (33.2)	22 (19.6)	50 (44.2)	40 (35.7)	< 0.001
T2, n (%)	73 (21.7)	16 (14.3)	29 (25.7)	28 (25.0)	0.067
C1, n (%)	145 (43.0)	28 (25.0)	65 (57.5)	52 (46.4)	< 0.001
C2, n (%)	22 (6.5)	5 (4.5)	7 (6.2)	10 (8.9)	0.395
**Follow-up**					
Follow-up interval (months)	42.96 (37.31–61.35)	47.60 (37.33–71.88)	42.10 (38.03–58.00)	45.23 (37.25–59.36)	0.184
Treated with steroids/other immunosuppressive agents, n (%)	181 (53.7)	54 (48.2)	52 (46.0)	75 (67.0)	0.003
**Outcome**					
Composite end point, n (%)	102 (30.3)	21 (18.8)	29 (25.7)	52 (46.4)	< 0.001
ESRD, n (%)	20 (5.9)	5 (4.5)	2 (1.8)	13 (11.6)	0.006

Values were presented as number (percent) or median (25th percentile-75th percentile). Kruskal–Wallis test or chi-square test was used to compare the values among groups. The last column represents the *P*-values from comparisons between group 1, 2 and 3.

*MAP* mean arterial pressure, *eGFR* estimated glomerular filtration rate, *ESRD* end stage renal disease.

### Renal ICR levels in IgAN, and clinical and histological features of patients stratified by ICR levels

**Figure 1:**
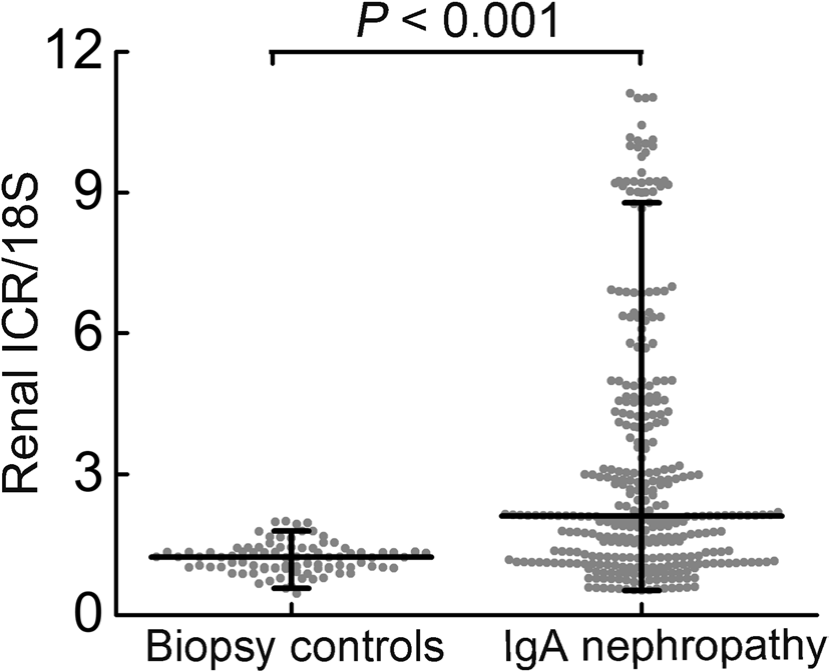
Renal ICR levels were significantly higher in patients with IgA nephropathy compared with biopsy controls. Renal ICR levels were detected by qPCR and normalized to 18S. IgA nephropathy, n = 337; Biopsy controls, n = 89. The horizontal lines from top down represent 75th percentile, median, and 25th percentile; the vertical lines represent interquartile range.

Renal ICR levels were significantly higher in IgAN patients at biopsy than in biopsy controls (median 2.11, IQR 1.21–4.27 *vs*. median 1.23, IQR 1.01–1.38, *P* < 0.001; Fig. 1). To explore the relationship between ICR levels and clinicopathological characteristics of IgAN patients, we divided the included patients into three groups based on the tertiles of ICR distributions (Table 1). The patients in group 3 had significantly higher MAP than those in groups 1 and 2 (*P* < 0.001). The proteinuria levels were higher in patients of group 3 than in group 1 (*P* = 0.001). Patients had gradually increased proportion of Oxford M1 score from group 1 to group 3 (*P* < 0.001). The proportion of Oxford S1, T1 and C1 score was lower in patients of group 1 than in groups 2 and 3 (*P* < 0.05). eGFR and the proportion of Oxford E1, T2 and C2 scores showed no significant differences among 3 groups. When compared with patients with Oxford T0 score, renal ICR levels were significantly elevated in patients with T1 or T2 (*P* < 0.001; Supplementary Fig. S1A). Renal ICR levels were significantly increased in patients with Oxford C1-2 compared with patients with C0 score (*P* < 0.01; Supplementary Fig. S1B). This indicated that increased renal ICR levels are associated with exacerbation of tubular atrophy/interstitial fibrosis and crescent formation.

### Association of renal ICR levels with disease progression in IgAN

**Figure 2:**
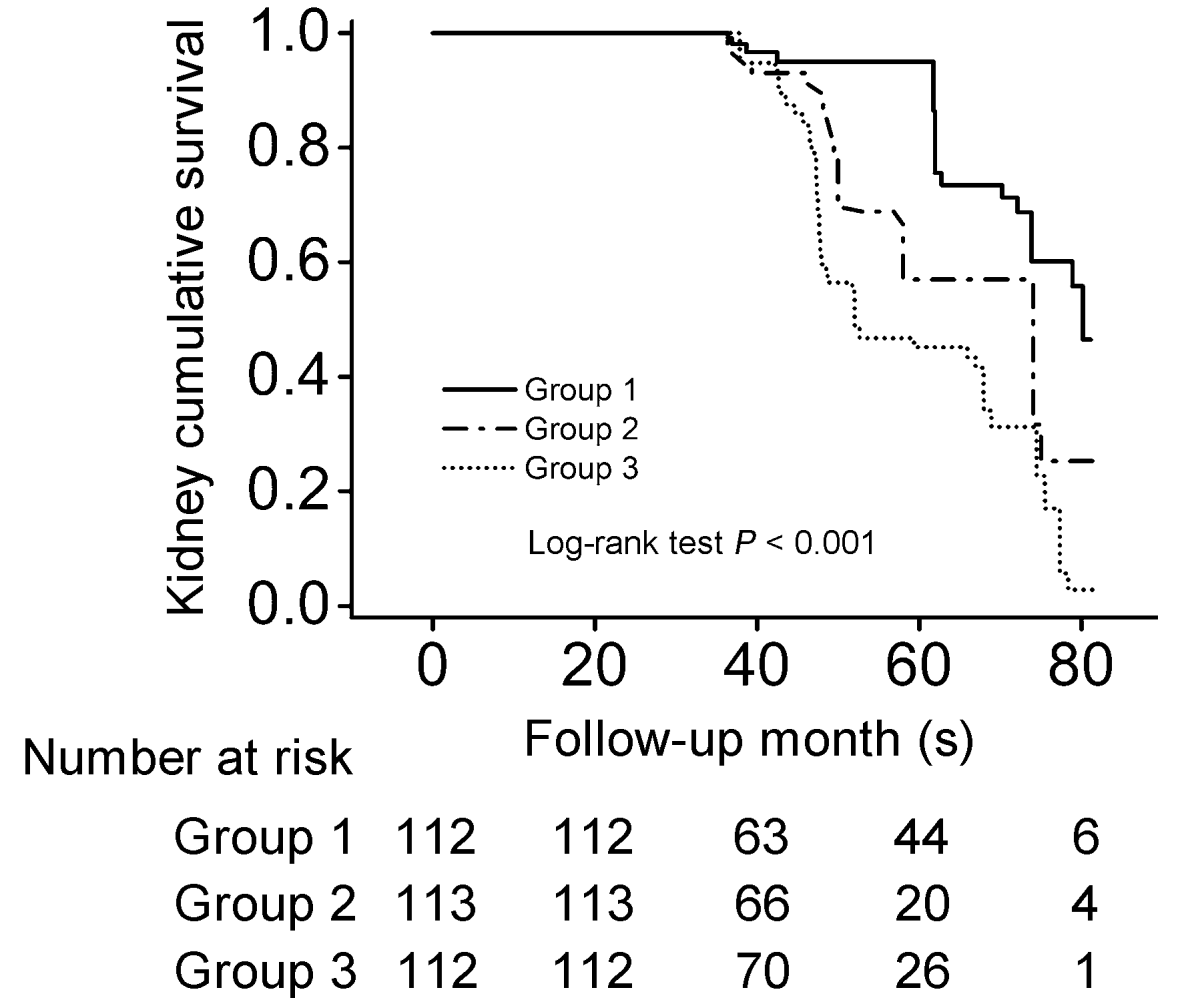
Kaplan–Meier kidney survival curves of patients with IgA nephropathy according to renal ICR tertiles. IgA nephropathy patients were stratified into three groups based on the tertiles of renal ICR levels: group 1 (< 1.56), group 2 (1.56–3.04), and group 3 (> 3.04).

We observed that the proportion of composite disease progression event was higher in patients in the highest tertile of renal ICR at biopsy (Group 3) compared with those in the lower 2 tertiles (Group 1 and 2; *P* < 0.05; Table 1). Consistently, Kaplan–Meier survival analysis revealed that the worse renal outcome was significantly associated with the higher tertile of renal ICR (*P* < 0.05; Fig. 2). We further evaluated the correlation between renal ICR level and IgAN progression using Cox proportional hazards models. In the univariate model, heavier proteinuria, higher Oxford M, S, T and C score, and higher tertile of renal ICR were all significantly associated with disease progression in IgAN (Supplementary Table S1). In the adjusted model, patients in the highest tertile of renal ICR had a greater risk for disease progression compared with those in the lowest tertile (hazard ratio [HR]: 3.526; 95% confidence interval [CI] 1.860–6.684; *P* < 0.001; Table 2 and Supplementary Table S1). The patients in the middle tertile of renal ICR had higher risk for IgAN progression after adjusting for clinical factors (model 2 HR: 2.733; 95% CI 1.464–5.101; *P* = 0.002; Table 2); however, adjusting for Oxford MEST-C scores and steroids or other immunosuppressive agents still observed increased risk but the confidence interval covered 1 (model 4 HR: 1.504; 95% CI 0.730–3.096; *P* = 0.268; Table 2). When expressed as a continuous variable, renal ICR levels were independently and significantly correlated with IgAN progression (HR:1.258; 95% CI 1.138–1.390; *P* < 0.001; Table 2). The sensitivity analysis in patients with proteinuria level < 1 g/24 h or ≥ 1 g/24 h found no significant interaction between levels of renal ICR and proteinuria on the IgAN progression (*P* for interaction > 0.05; Supplementary Table S2).

**Table 2:** Associations of renal ICR level with disease progression in IgA nephropathy patients.

	Renal ICR levels Median (IQR)	Unadjusted HR (95%CI) and *P* value	Adjusted HR (95% CI) and *P* value			
			Model 1	Model 2	Model 3	Model 4
ICR (continuous trait)	2.11 (1.21–4.27)	1.290 (1.197–1.392) 0.000	1.292 (1.197–1.395) 0.000	1.340 (1.228–1.463) 0.000	1.239 (1.124–1.366) 0.000	1.258 (1.138–1.390) 0.000
**ICR tertiles**						
Group 1 1st tertile	1.07 (0.77–1.21)	Reference	Reference	Reference	Reference	Reference
Group 2 2nd tertile	2.11 (1.87–2.56)	2.491 (1.407–4.411) 0.002	2.203 (1.238–3.921) 0.007	2.733 (1.464–5.101) 0.002	1.770 (0.869–3.604) 0.116	1.504 (0.730–3.096) 0.268
Group 3 3rd tertile	5.74 (4.27–9.01)	4.025 (2.405–6.737) < 0.001	3.943 (2.342–6.638) < 0.001	5.656 (3.184–10.044) < 0.001	3.455 (1.808–6.603) < 0.001	3.526 (1.860–6.684) < 0.001
*P* for trend		< 0.001	< 0.001	< 0.001	< 0.001	< 0.001

Model 1 adjusted for sex (analyzed as dichotomous data) and age. Model 2 adjusted for covariates in model 1 plus estimated glomerular filtration rate, proteinuria, and mean arterial pressure. Model 3 adjusted for covariates in model 2 plus Oxford MEST-C score. Model 4 adjusted for covariates in model 3 plus steroids or other immunosuppressive agents (yes or no, analyzed as dichotomous data). The disease progression event was defined as end-stage renal disease or ≥ 40% decline in estimated glomerular filtration rate. *P* values for trends were calculated by entering the median value of each tertile of renal ICR level as a continuous variable.

*IQR* interquartile range, *HR* hazard ratio, *CI* confidence interval.

### Increment prognostic value of renal ICR

Survival analysis found a trend of improved risk prediction of IgAN progression by adding renal ICR to the conventional model containing traditional risk factors (C statistic, 0.79 [95% CI 0.75–0.84] vs. 0.77 [95% CI 0.72–0.82]). Meanwhile, the addition of renal ICR to the conventional model significantly improved risk reclassification for the composite disease progression event at 5 years as assessed by both the net reclassification index (NRI, 0.31 [95% CI 0.01–0.50]) and the integrated discrimination index (IDI, 0.10 [95% CI 0.04–0.16]).

### ICR is upregulated in the TGF-β1-induced HK-2 cells

**Figure 3:**
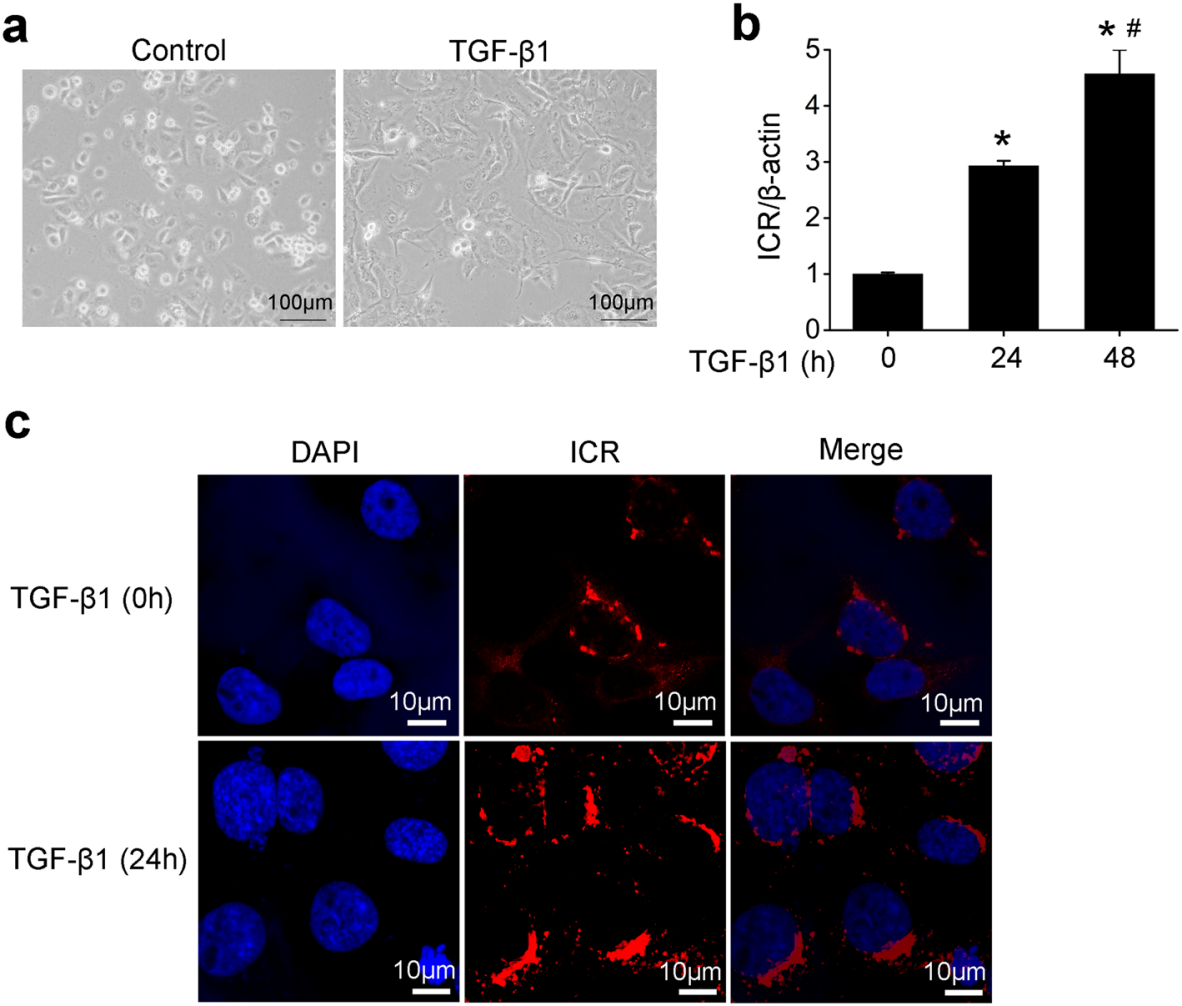
ICR was upregulated in human proximal tubular HK-2 cells cultured with TGF-β1. (**a**) The cell morphology of HK-2 cells treated with or without TGF-β1 (10 ng/ml) for 48 h. (**b**) QPCR analysis after normalization against β-actin showed an increase in the level of ICR in HK-2 cells treated with TGF-β1(10 ng/ml, 24 h and 48 h) compared to the cells without treatment. (**c**) Representative confocal fluorescence in situ hybridization images showed that ICR was mainly distributed in cytoplasm of HK-2 cells treated with or without TGF-β1 (10 ng/ml) for 24 h. Data are presented as means ± SD of three independent experiments. *P (vs. TGF-β1 0 h) < 0.05, ^#^*P* (vs. TGF-β1 24 h) < 0.05.

TGF-β1 stimulates profibrotic changes in proximal tubular cells and plays a critical role in the pathogenesis of tubulointerstitial fibrosis^19^. In the current study, we found that HK-2 cells displayed a spindle-shaped, fibroblast-like morphology 48 h after TGF-β1 treatment (Fig. 3a). The expression of ICR was significantly upregulated in HK-2 cells treated with TGF-β1 for both 24 h and 48 h (Fig. 3b). Furthermore, ICR fluorescence in situ hybridization (FISH) staining of HK-2 cells revealed that ICR was mainly located in the cytoplasm both in the presence or absence of TGF-β1 (Fig. 3c).

### Knockdown of ICR suppresses fibrotic changes along with decreased phosphorylation of Akt and mTOR in vitro

**Figure 4:**
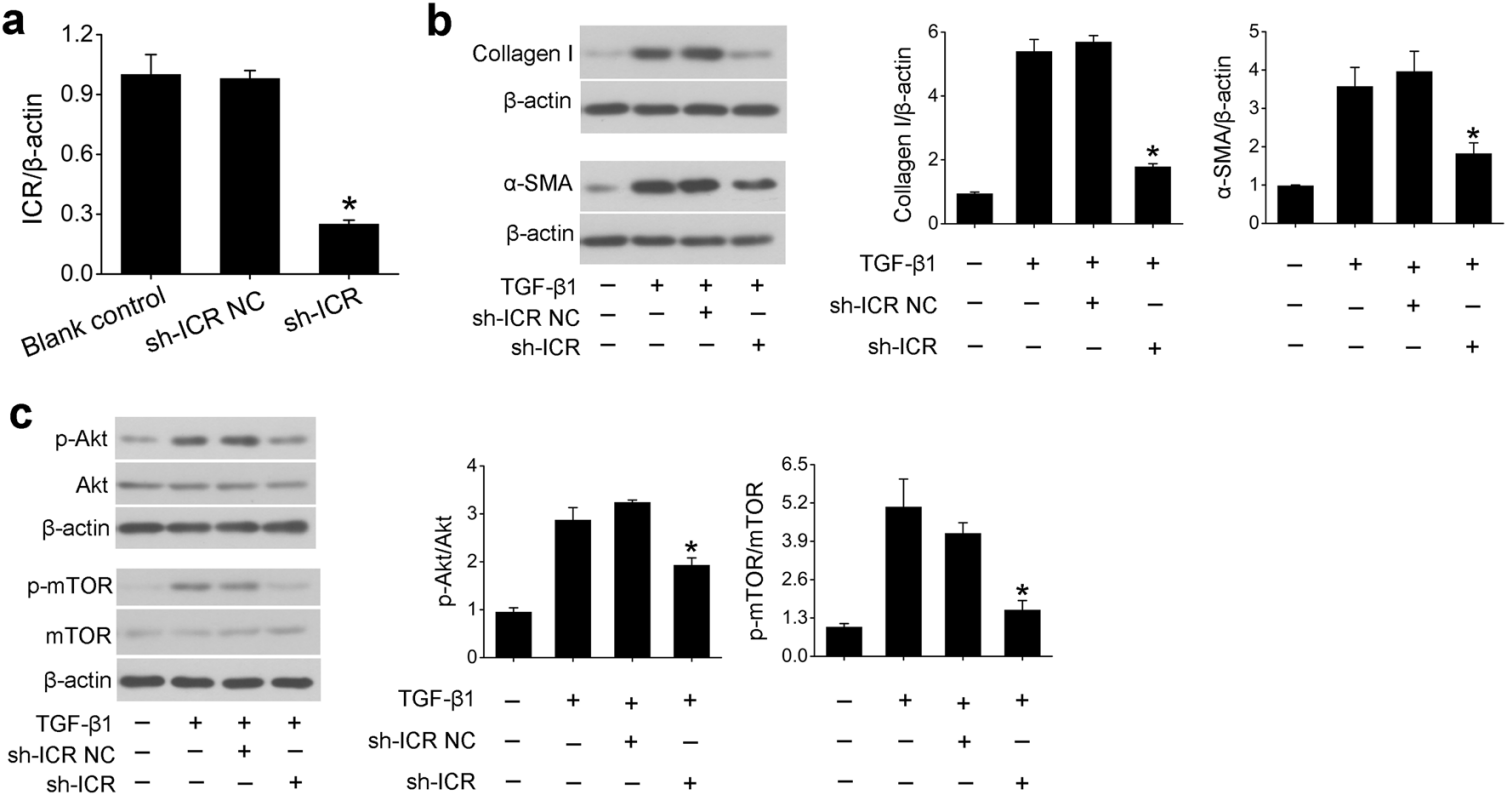
ICR downregulation attenuates fibrotic changes and inhibits phosphorylation of Akt and mTOR in TGF-β1-treated human proximal tubular HK-2 cells. (**a**) QPCR analysis normalized to β-actin showed a significant decrease in the level of ICR in HK-2 cells transfected with ICR shRNA plasmids (sh-ICR) compared with its negative control (sh-ICR NC). Knockdown of ICR upon transfection with sh-ICR plasmid significantly reduced both profibrotic protein levels of collagen I and α-SMA (**b**), and phosphorylation of Akt and mTOR (**c**) in HK-2 cells stimulated with TGF-β1 (10 ng/ml, 48 h). The protein levels of collagen I and α-SMA were normalized to β-actin; the phosphorylated Akt and mTOR were normalized to Akt and mTOR, respectively. The original blots are presented in Supplementary Figs. S2 and Fig. S3. Data are presented as means ± SD of three independent experiments. *P < 0.05, significantly different from negative control group (sh-ICR NC or TGF-β1 + sh-ICR NC).

To further evaluate the effects of ICR on fibrotic changes, we first transfected HK-2 cells with plasmid containing ICR shRNA (sh-ICR) or its negative control (Fig. 4a). The results showed that ICR levels were decreased by 75% in HK-2 cells transfected with sh-ICR plasmid compared with its negative control. Importantly, transfection with sh-ICR plasmid markedly reduced the levels of profibrotic protein collagen I and α-SMA in HK-2 cells treated with TGF-β1 (Fig. 4b). Previous studies have demonstrated that activation of Akt/mTOR signaling pathway is involved in renal fibrosis^20, 21^. Consistently, we observed increased phosphorylation of Akt at Ser-473 and mTOR at Ser-2448 in TGF-β1-treated HK-2 cells (Fig. 4c). However, when ICR was downregulated by transfection of sh-ICR plasmid, we found significantly decreased phosphorylation both at Ser-473 in Akt and at Ser-2448 in mTOR in HK-2 cells upon TGF-β1 treatment (Fig. 4c). These findings indicate that inhibition of ICR attenuates fibrotic changes in TGF-β1-induced HK-2 cells and that this regulatory effect of ICR on fibrosis is associated with phosphorylation of Akt and mTOR.

## Discussion

The present study clearly shows a significant increase in ICR levels in kidney tissue from patients with IgAN. Moreover, multivariable Cox regression analyses revealed that renal ICR levels were independently and positively correlated with disease progression in IgAN after adjusting for traditional risk factors, including age, sex, MAP, eGFR, proteinuria, pathological parameters of the Oxford classification and immunosuppressant therapy. Importantly, the addition of renal ICR level to the traditional risk factors significantly improved the risk prediction of IgAN progression. We further showed that inhibition of ICR attenuated the fibrotic changes in TGF-β1-treated renal proximal tubule cells associated with reduced phosphorylation of Akt and mTOR in vitro. These findings suggest that renal ICR will be a promising predictor for disease progression in IgAN and provide new insights into mechanism of proximal tubule injury in condition with altered levels of renal ICR.

ICR was firstly identified to regulate ICAM-1 expression and promote hepatocellular carcinoma metastasis by binding to ICAM-1 mRNA and increasing its stability^16^. Additionally, ICR has been observed to play key roles in retinal vascular diseases and allergic asthma^15, 22^. In this study, ICR was upregulated in kidney biopsy tissue from patients with IgAN. Moreover, the patients in the highest tertile of renal ICR had higher proteinuria level and higher proportion of Oxford M1, S1, T1, and C1 scores compared with those in the lowest tertile. The proportion of Oxford S1, T1, and C1 scores in patients of the middle tertile were comparable to those of the highest tertile. However, no significant differences in proportion of Oxford T2 and C2 scores were observed among patients in the three tertiles of renal ICR, suggesting that ICR may not play a key role in the Oxford T2 and C2 lesions. The renal ICR levels were increased with exacerbation of tubulointerstitial injury (from T0 to T1 or T2 score) and crescent formation (from C0 to C1-2 score). Future study is needed to explore the mechanism underlying associations of the clinicopathological parameters (e.g., proteinuria, eGFR, and Oxford MEST-C scores) and renal ICR levels in IgAN.

Notably, in our study, higher tertile of renal ICR is associated with higher proportion of composite disease progression event in IgAN. Consistently, Kaplan–Meier survival curves showed that the renal outcome became worse according to the ascending tertiles of renal ICR levels in IgAN. More importantly, renal ICR has been found to be an independent risk factor for disease progression in IgAN patients after adjusting for traditional risk factors. These findings are in accordance with the results from the sensitive analysis in subgroup stratified by proteinuria level at biopsy. In addition, we observed a trend of increased C statistics value, and a significant increase in NRI as well as IDI value after adding renal ICR level to the model containing traditional risk factors. This supports the notion that renal ICR would be a useful biomarker in predicting the risk for poor renal outcome in IgAN. We have not directly shown the localization of ICR in kidney tissue from the IgAN patients. Since the paraffin-embedded renal biopsy sections are relatively old and ICR in the sections tends to degrade easily, it is unlikely to detect ICR by in situ* hybridization* successfully in those patients. Previous studies with regard to lncRNA measurement have mostly used the qPCR technique to analyze expression of lncRNA and associations of lncRNA with relevant diseases, which is in line with our study ^23–25^. The future experimental work is needed to confirm this.

Furthermore, we found that inhibition of ICR obviously alleviated fibrotic changes in proximal tubular cells upon TGF-β1 treatment in vitro. It is well known that renal fibrosis is the major pathological mechanism of CKD. Thus, in addition to IgAN, modulating ICR expression is likely to be a relevant target for preventing or attenuating progression of other chronic kidney diseases with renal fibrosis and increased levels of ICR. Akt activation has been widely reported to participate in TGF-β1-induced renal epithelial-mesenchymal transition leading to renal fibrosis^26^. The activation of Akt can further induce the activation of mTOR, which stimulates protein synthesis for cell growth and proliferation. Accordingly, we detected that TGF-β1 treatment caused an increase in phosphorylation of Akt at Ser-473 and mTOR at Ser-2448 in proximal tubular cells in vitro*,* and inhibition of ICR significantly reduced this phosphorylation. Our findings indicate that the underlying mechanism of suppressing fibrotic changes by regulating ICR may be associated with inhibition of Akt/mTOR signaling pathway which is involved in IgAN ^27, 28^. Further studies, including the use of animal models in vivo, are required to confirm this potential mechanism of increased ICR expression and renal fibrosis.

There are several limitations in the current study. First, this was a single-center study and validation studies from other population are warranted. Second, in-depth molecular experiments, including the investigation of potential role of ICR in regulating ICAM-1 expression, are needed with regard to mechanism of ICR in renal fibrosis. It would be better to measure ICR in an easily accessible medium like serum and urine. Unfortunately, those samples from the patients are unavailable now. There may be some clinical implications, which is required to explore in the future. Nevertheless, our study may be considered a proof of concept of a new regulatory mechanism in renal fibrosis and propose a novel role of lncRNA within the kidney.

In conclusion, our study strongly supports an important role of renal ICR in the prediction of disease progression in IgAN independently of clinical and pathological characteristics. The addition of renal ICR level to the traditional risk factors improved the risk assessment of IgAN progression. Moreover, inhibition of ICR would attenuate fibrotic changes in TGF-β1-induced proximal tubular cells potentially through Akt/mTOR signaling pathway, which points to a novel anti-fibrotic treatment in renal diseases. Further experimental work is required to explore this mechanism in more detail.

## Methods

### Patients and samples

We conducted a retrospective cohort study of 413 patients with IgAN confirmed by kidney biopsy at the First Affiliated Hospital of Zhengzhou University between May 2013 and March 2018. All of these patients had additional biopsy specimens for this research. We excluded 26 patients with missing baseline clinical data, 29 with missing follow-up data, and 21 with missing treatment regimens. All recruited patients were followed up for at least 3 years. The patients with secondary IgAN, including IgA vasculitis, hepatitis B virus associated glomerulonephritis, liver cirrhosis, systemic lupus erythematosus, were excluded. Therefore, the final cohort included 337 patients with primary IgAN. Kidney biopsy specimens were collected from the included patients along with the clinical, kidney biopsy, and were immediately frozen at − 80 °C until measurement. Moreover, normal kidney tissue from nephrectomy specimens of 89 age- and sex-matched patients with renal cell carcinoma served as biopsy controls (Supplementary Table S3).

This study was conducted in compliance with the Declaration of Helsinki, and was approved by the ethics committee of the First Affiliated Hospital of Zhengzhou University, China (Approval No: 2019-KY-361). Informed consent was obtained from all recruited participants.

### Clinical and histological manifestations

Clinical data, including sex, age, MAP, serum creatine, and 24 h urinary protein excretion, were collected at the time of kidney biopsy (defined as baseline). The treatment regimens of patients, including the use of steroids or other immunosuppressant, were recorded during the follow-up. The eGFR was calculated using the CKD-epidemiology Collaboration formula^29^. All kidney biopsy sections were reviewed by two independent pathologists who were blinded to patients’ clinical data. The pathological lesions were evaluated according to the Oxford classification (MEST-C score)^30^. Briefly, it includes 4 parameters as follows: mesangial hypercellularity (M0/M1), endocapillary hypercellularity (E0/E1), segmental sclerosis (S0/S1), interstitial fibrosis/tubular atrophy (T0/T1/T2), and cellular/fibrocellular crescents (C0/C1/C2).

ESRD event was defined as eGFR < 15 mL/min/1.73 m^2^ or need for renal replacement therapy. The composite disease progression event was defined as a permanent reduction ≥ 40% in eGFR over baseline or ESRD, whichever occurred first. Those two indicated outcomes were confirmed by a second evaluation at least 4 weeks later.

### Cell culture and transfection

The HK-2 cell is an immortalized human proximal tubule cell line, and obtained from Wanleibio (Shenyang, China) and cultured in DMEM (Lonza, Basel, Switzerland) supplemented with 10% FBS, 100 U/ml penicillin, and 0.1 mg/ml streptomycin. The cells were incubated at 37 °C and 5% CO_2_ in a humidified atmosphere. When cells confluency reached about 60%, the growth medium was replaced with serum-free medium for another 12 h incubation. After that, cells were incubated with 10 ng/ml TGF-β1 for 24 h or 48 h. The plasmids containing ICR shRNA were designed to knockdown of ICR expression and purchased from Wanleibio. HK-2 cells were seeded in 6-well plate 24 h before transfection. Cells were transfected with sh-ICR plasmid or its negative control using Lipofectamine 3000 transfection reagent according to the manufacturer’s instructions. QPCR was used to evaluate the transfection efficiency of sh-ICR plasmid 24 h after transfection. These transfected cells were then stimulated with TGF-β1 for another 48 h incubation. The cellular morphology images were obtained with a Leica DMR microscope (Leica Microsystems, Wetzlar, Germany).

### Fluorescence in situ hybridization

Fluorescence in situ hybridization was performed with an anti-ICR oligonucleotide probe labeled with Cy3. Cells were fixed in 4% paraformaldehyde, dehydrated by ethanol, and then incubated with ICR probe in the dark overnight at 37 °C. The next day, the cell nuclei were counterstained with DAPI. A confocal microscope (Carl Zeiss, Oberkochen, Germany) was used to capture the images.

### Western blot analysis

Cellular proteins were obtained by lysing the cells in lysis buffer containing 1% Triton X-100, 150 mM NaCl, and 50 mM Tris·HCl. Proteins extracted from cells were separated by SDS-PAGE and analyzed by Western blot as previously described^31^. The following antibodies purchased from Wanleibio were used: AKT antibody (1: 500, WL0003b), p-AKT antibody (1: 500, WLP001a), mTOR antibody (1: 500, WL02477), p-mTOR antibody (1: 500, WL03694), collagen I antibody (1: 500, WL0088), α-SMA antibody (1: 500, WL02510), and β-actin antibody as loading control (1: 1000, WL01372). Horseradish peroxidase-conjugated sheep anti-rabbit immunoglobulins (WLA023) were used for detection with ECL substrate. ImageJ 1.47 Software was used to quantify the signal.

### Total RNA extraction and qPCR analysis

The amounts of ICR in kidney tissue from patients were measured using qPCR by KangChen Bio-tech (Shanghai, China)^32^. Briefly, total RNA was purified from kidney tissue using TRIzol^®^ Reagent (Thermo Fisher Scientific, Waltham, USA). Then 20 μl reaction including 1.5 μg total RNA and 1 µl (0.5 µg/µl) Oligo(dT)_18_ primer was used for reverse transcription. QPCR was run on the QuantStudio™ 5 Real-Time PCR System (Applied Biosystems, Foster, USA). For qPCR analysis in HK-2 cells, total RNA was purified from cells by TRIpure (BioTeke, Beijing, China). Complementary DNA was produced by RNA and qPCR was performed with SYBR Green. The expression of ICR in kidney tissue and cells were normalized to 18S rRNA and β-actin, respectively. The relative changes in ICR were calculated using the 2^−ΔΔCt^ method, where ΔCt = Ct_ICR_ − Ct_18S/β-actin_ and ΔΔCt = ΔCt _experimental_ − ΔCt _control_. Primers purchased from KangChen Bio-tech were as follows: ICR: sense: 5′-CGCTCAGCCGGCATAGAACA-3′, antisense: 5′-TCGGTGAGGCACCCCTGTAA-3′; 18S: sense: 5′-CAGCCACCCGAGATTGAGCA-3′, antisense: 5′-TAGTAGCGACGGGCGGTGTG-3′; β-actin: sense: 5′-GGCACCCAGCACAATGAA-3′, antisense: 5′-TAGAAGCATTTGCGGTGG-3′.

### Statistical analysis

The quantitative data were expressed as means ± SD or median (IQR) and compared using the t-test, Mann–Whitney U-test or Kruskal–Wallis test, as appropriate. Categorical data were expressed as frequencies and percentages and compared by chi-square test. The patients were divided into 3 groups by tertiles of renal ICR level (group 1, < 1.56; group 2, 1.56–3.04; group 3, > 3.04). We used the Kaplan–Meier analysis to produce cumulative kidney survival curves, and used the log-rank test to analyze differences between the curves. The unadjusted and multivariable-adjusted Cox proportional hazards models were applied to evaluate the effect of renal ICR on risk for disease progression in IgAN. In the models, renal ICR was analyzed as either 3-level categorical variable defined by tertiles of renal ICR level, or a continuous variable. The results were expressed as HR and 95% CI. *P*-values for trends in the Cox proportional hazards models were calculated by entering the median value of each tertile of renal ICR level as a continuous variable. We used interaction terms to test the interaction between renal ICR level and proteinuria level on disease progression in IgAN. Furthermore, C statistic, NRI and IDI were calculated to assess the incremental prognostic value of renal ICR for 5-year kidney survival outcome after biopsy beyond traditional risk factors^33, 34^. Statistical analysis was performed using the SPSS software (version 19.0; IBM Corp., Armonk, USA) and R version 4.0.2. A *P*-value < 0.05 was considered statistically significant.

## Supplementary Information

Supplementary Information.

## Data Availability

All data are available from the corresponding author upon reasonable request.
