# Supplementary material for: ICAM-1 related long noncoding RNA is associated with progression of IgA nephropathy and fibrotic changes in proximal tubular cells
Source: Sci Rep. 2022 Jun 10;12:9645. doi: 10.1038/s41598-022-13521-6 (PMC9187724; doi:10.1038/s41598-022-13521-6)
Supplement: Supplementary file 1 — Supplementary Information. [file 41598_2022_13521_MOESM1_ESM.docx]

**
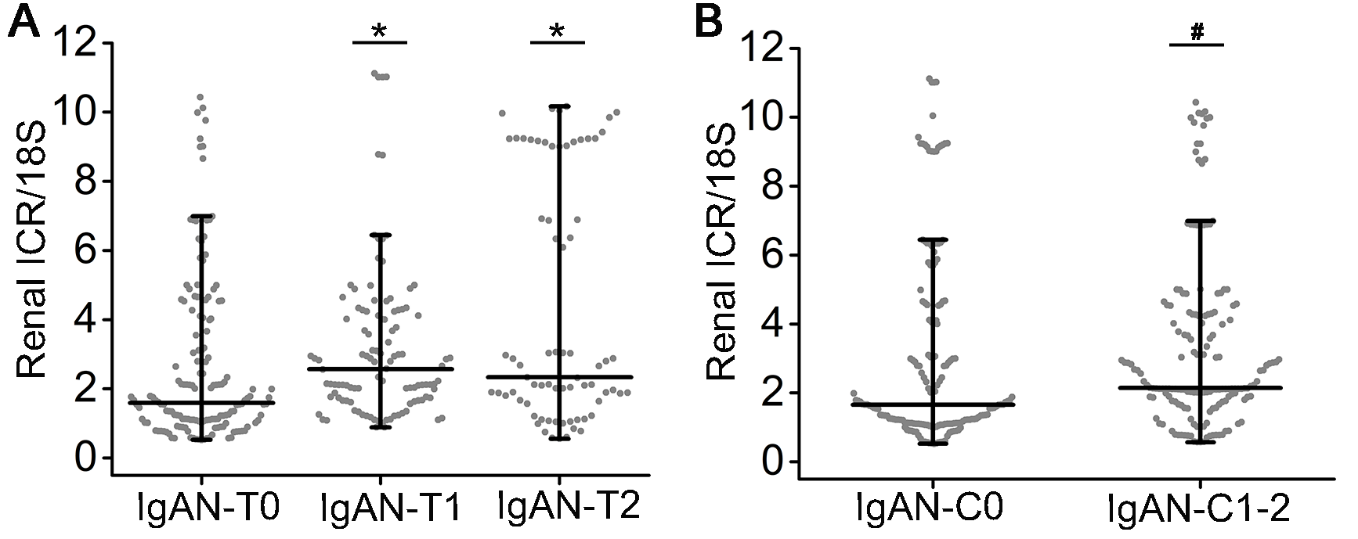
**

**Supplementary Figure S1.** (A) Renal ICR levels were significantly increased in IgAN patients with T1 or T2 when compared with patients with T0. (B) The IgAN patients with C1-2 had significantly higher renal ICR levels as compared with patients with C0. Renal ICR levels were detected by qPCR and normalized to 18S. IgAN-T0, IgAN patients with T0, n = 152; IgAN-T1, IgAN patients with T1, n = 112; IgAN-T2, IgAN patients with T2, n = 73; IgAN-C0, IgAN patients with C0, n = 170; IgAN-C1-2, IgAN patients with C1-2, n = 167. IgAN, IgA nephropathy. The horizontal lines from top down represent 75th percentile, median, and 25th percentile; the vertical lines represent interquartile range. **P* < 0.001 (vs. IgAN-T0), ^#^*P*< 0.01 (vs. IgAN-C0).

|  | Univariate | | Multivariate | |
| --- | --- | --- | --- | --- |
| Characteristics | HR (95%CI) | *P* | HR (95%CI) | *P* |
| Age (years) | 0.998(0.981-1.015) | 0.816 | 1.016(0.992-1.040) | 0.199 |
| Sex | 0.510(0.337-0.772) | 0.001 | 0.378(0.216-0.662) | 0.001 |
| MAP (mmHg) | 1.000(0.982-1.019) | 0.988 | 0.993(0.967-1.019) | 0.595 |
| eGFR (mL/min/1.73m^2^) | 0.996(0.989-1.003) | 0.290 | 0.987(0.977-0.997) | 0.015 |
| Proteinuria (g/24h) | 1.316(1.095-1.582) | 0.003 | 1.263(0.937-1.703) | 0.125 |
| Oxford classification |  |  |  |  |
| M1 | 1.889(1.275-2.797) | 0.002 | 1.130(0.712-1.794) | 0.603 |
| E1 | 1.157(0.773-1.732) | 0.479 | 0.913(0.545-1.527) | 0.727 |
| S1 | 2.273(1.502-3.438) | <0.001 | 1.226(0.655-2.295) | 0.524 |
| T1 | 2.285(1.346-3.878) | 0.002 | 2.339(1.277-4.285) | 0.006 |
| T2 | 5.224(3.079-8.862) | <0.001 | 4.926(2.631-9.222) | <0.001 |
| C1 | 2.439(1.545-3.852) | <0.001 | 2.373(1.303-4.319) | 0.005 |
| C2 | 2.878(1.686-4.912) | <0.001 | 3.006(1.552-5.823) | 0.001 |
| Treated with steroids/other immunosuppressive agents | 1.213(0.815-1.806) | 0.341 | 0.484(0.244-0.963) | 0.039 |
| Renal ICR |  |  |  |  |
| Group 1 (1st tertile) | Reference |  | Reference |  |
| Group 2 (2nd tertile) | 2.491(1.407-4.411) | 0.002 | 1.504(0.730-3.096) | 0.268 |
| Group 3 (3rd tertile) | 4.025(2.405-6.737) | <0.001 | 3.526(1.860-6.684) | 0.000 |

**Supplementary** **Table S1.** Unadjusted and multivariable-adjusted Cox regression models evaluating associations between renal ICR level and disease progression in IgA nephropathy patients. MAP, mean arterial pressure; eGFR, estimated glomerular filtration rate; HR, Hazard ratio; CI, confidence interval.

|  | Events  n (%) | Unadjusted  HR (95%CI) and *P* value | Adjusted HR (95%CI) and *P* value | | |
| --- | --- | --- | --- | --- | --- |
|  |  |  | Model 1 | Model 2 | Model 3 |
| Proteinuria < 1g/d |  |  |  |  |  |
| Group 1  1st tertile  (*n*=42) | 5(11.9) | Reference | Reference | Reference | Reference |
| Group 2  2nd tertile  (*n*=40) | 16(40) | 3.314(1.209-9.081)  0.020 | 1.466(0.433-4.967)  0.539 | 0.135(0.016-1.110)  0.062 | 0.125(0.011-1.360)  0.088 |
| Group 3  3rd tertile  (*n*=33) | 18(54.5) | 4.631(1.709-12.552)  0.003 | 2.900(1.010-8.323)  0.048 | 3.449(0.608-19.559)  0.162 | 3.466(0.607-19.779)  0.162 |
| *P* for trend |  | 0.003 | 0.014 | 0.089 | 0.136 |
| Proteinuria ≥ 1g/d |  |  |  |  |  |
| Group 1  1st tertile  (*n*=70) | 16(22.9) | Reference | Reference | Reference | Reference |
| Group 2  2nd tertile  (*n*=73) | 13(17.8) | 2.377(1.079-5.238)  0.032 | 2.197(1.003-4.815)  0.049 | 1.551(0.614-3.922)  0.353 | 1.369(0.547-3.429)  0.502 |
| Group 3  3rd tertile  (*n*=79) | 34(43.0) | 3.623(1.932-6.794)  0.000 | 3.882(2.052-7.342)  0.000 | 5.018(2.242-11.229)  0.000 | 5.794(2.479-13.539)  0.000 |
| *P* for trend |  | <0.001 | <0.001 | <0.001 | <0.001 |

**Supplementary Table S2.** Associations of renal ICR with disease progression in IgA nephropathy patients stratified by baseline proteinuria. HR, hazard ratio; CI, confidence interval. Model 1 adjusted for sex (analyzed as dichotomous data) and age. Model 2 adjusted for covariates in model 1 plus estimated glomerular filtration rate, mean arterial pressure, and Oxford MEST-C score. Model 3 adjusted for covariates in model 2 plus steroid or other immunosuppressive agents (yes or no, analyzed as dichotomous data). The disease progression event was defined as end-stage renal disease or ≥ 40% decline in estimated glomerular filtration rate. *P* values for trends were calculated by entering the median value of each tertile of renal ICR level as a continuous variable.

**
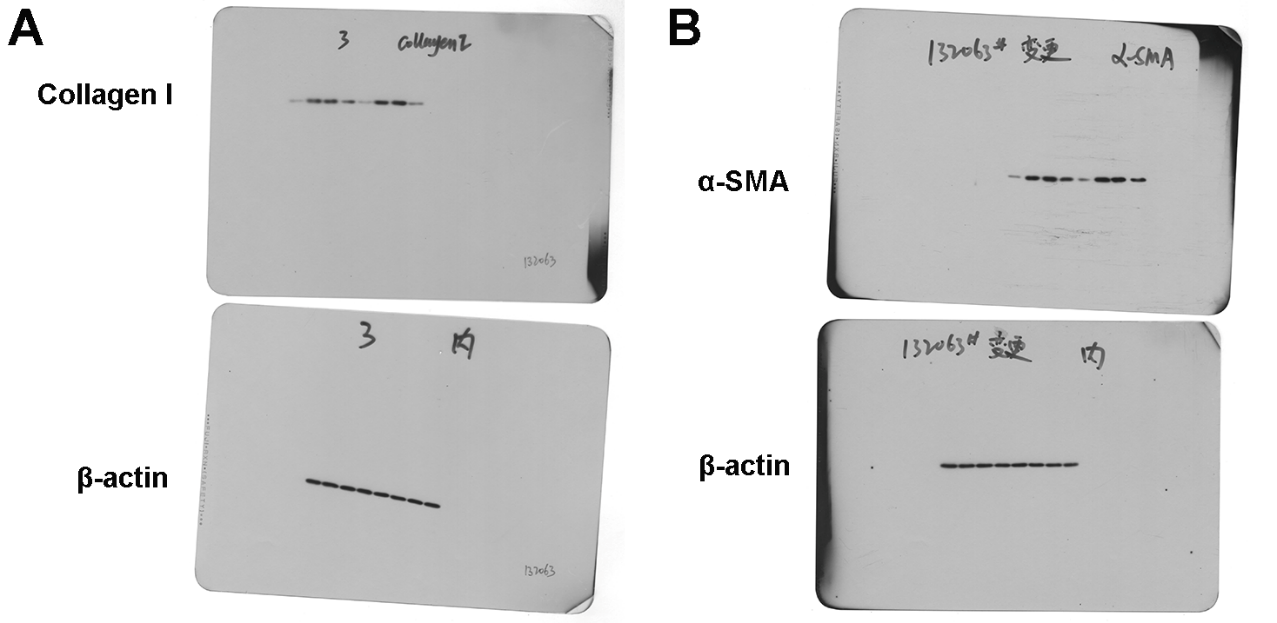
**

**Supplementary Figure S2.** The original blots of Figure 4b in the main text. (A-B) The last 4 columns were presented in the main text.

**
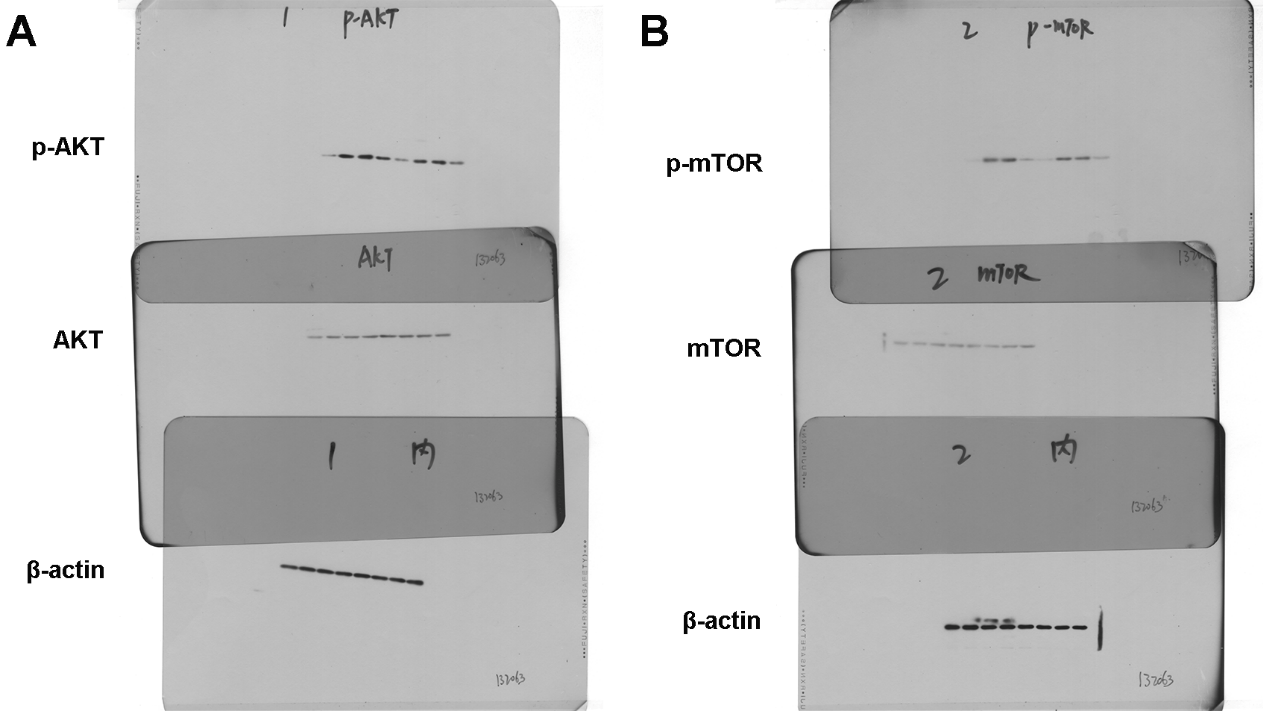
**

**Supplementary Figure S3.** The original blots of Figure 4c in the main text. (A-B) The last 4 columns were presented in the main text.

| Variables | Biopsy controls  (*n* = 89) | IgA nephropathy  (*n* = 337) | *P* |
| --- | --- | --- | --- |
| Age (years) | 37.0 (31.5-44.0) | 35.0 (26.5-44.0) | 0.061 |
| Sex (men, %) | 56 (62.9) | 193 (57.3) | 0.336 |
| eGFR (mL/min/1.73m^2^) | 108.0 (88.8-117.0) | 83.2 (58.2-108.2) | < 0.001 |
| Proteinuria (g/24h) | 0.08 (0.04-0.14) | 1.48 (0.83-2.77) | < 0.001 |

**Supplementary Table S3.** Demographic and clinical data of biopsy controls and IgA nephropathy patients. eGFR, estimated glomerular filtration rate. Values were presented as number (percent) or median (25th percentile-75th percentile). Mann-Whitney U-test or chi-square test was used to compare the values between groups.
